# Vancomycin resistant enterococci (VRE) in Swedish sewage sludge

**DOI:** 10.1186/1751-0147-51-24

**Published:** 2009-05-29

**Authors:** Leena Sahlström, Verena Rehbinder, Ann Albihn, Anna Aspan, Björn Bengtsson

**Affiliations:** 1 Finnish Food safety Authority, Evira, Mustialankatu 3, 00790 Helsinki, Finland; 2 National Veterinary Institute, 75189 Uppsala, Sweden

## Abstract

**Background:**

Antimicrobial resistance is a serious threat in veterinary medicine and human healthcare. Resistance genes can spread from animals, through the food-chain, and back to humans. Sewage sludge may act as the link back from humans to animals. The main aims of this study were to investigate the occurrence of vancomycin resistant enterococci (VRE) in treated sewage sludge, in a Swedish waste water treatment plant (WWTP), and to compare VRE isolates from sewage sludge with isolates from humans and chickens.

**Methods:**

During a four month long study, sewage sludge was collected weekly and cultured for VRE. The VRE isolates from sewage sludge were analysed and compared to each other and to human and chicken VRE isolates by biochemical typing (PhenePlate), PFGE and antibiograms.

**Results:**

Biochemical typing (PhenePlate-FS) and pulsed field gel electrophoresis (PFGE) revealed prevalence of specific VRE strains in sewage sludge for up to 16 weeks. No connection was found between the VRE strains isolated from sludge, chickens and humans, indicating that human VRE did not originate from Swedish chicken.

**Conclusion:**

This study demonstrated widespread occurrence of VRE in sewage sludge in the studied WWTP. This implies a risk of antimicrobial resistance being spread to new farms and to the society via the environment if the sewage sludge is used on arable land.

## Background

Enterococci are naturally occurring bacteria in the intestinal tract of humans and animals, and are often used as indicators of faecal contamination in water [1]. Enterococci are resistant to environmental stress and may persist for a long time outside their hosts. They are not considered severe pathogenic organisms, but some species, e.g. *Enterococcus (E.) faecalis *and *E. faecium*, are important causes of nosocomial infections [2,3]. Antimicrobial resistance in strains causing nosocomial infections is a growing problem and vancomycin resistant enterococci (VRE) in particular are considered a serious threat in hospitals around the world [4]. Vancomycin is often used as a last resort in treatment of antibiotic resistant gram-positive bacterial infections caused by organisms such as multi-resistant enterococci and methicillin resistant staphylococci. In the USA, the prevalence of VRE is mainly documented as nosocomial infection in humans [5,6]. In Europe, nosocomial infections with VRE are less common, but such bacteria are widespread among healthy livestock [5] as a consequence of previous use of avoparcin (an analogue to vancomycin) as a feed additive in animal husbandry [7-9].

Vancomycin resistance in nosocomial isolates of enterococci is usually mediated by the resistance genes *vanA *or *vanB *[4]. High-level vancomycin resistance (MIC >64 mg/L) is mediated by the *vanA *gene cluster, located on the transferable genetic element transposon Tn*1546 *[10]. Variable levels of vancomycin resistance (MIC 4–1000 mg/L) characterise the *vanB *genotype and the gene cluster is located on another mobile genetic element, Tn*1547 *[10]. Some enterococci (including *E. gallinarum*) may posses intrinsic, but not transferable, resistance against vancomycin, coded by *vanC *(MIC 2–32 mg/L) [10].

Vancomycin resistance may be spread either by clonal dissemination of resistant isolates or by transfer of the resistance genes to other strains of the same bacterial species or to other species or genera (horizontal gene transfer). Interspecies transfer of transposable (Tn*1546*-like) genetic elements between different species of enterococci [11] and from enterococci to *Listeria monocytogenes *[12] have been demonstrated. The first documented case of horizontal transfer of the *vanA *gene to the most feared cause of nosocomial infections, *Staphylococcus aureus*, was documented in 2002 in the USA [13]. In addition, Marcineck et al. [14] demonstrated gene transfer between different strains of *E. faecalis *in the natural environment of a waste water treatment plant. Moreover, *E. faecium vanA *genetic elements of animal origin have recently been proven capable of transferring to *E. faecium *strains of human origin in the intestine of human volunteers [15].

Iversen et al. [16] demonstrated 60% prevalence of VRE in samples of raw sewage from Swedish waste water treatment plants (WWTP) and 19% prevalence in treated sewage. These levels are surprisingly high, since nosocomial VRE infections are uncommon in Sweden [17] and the presence of VRE in healthy humans is considered rare [18]. Moreover, vancomycin is seldom used in routine human healthcare in Sweden [17]. Likewise, VRE are rare in Swedish livestock production and avoparcin has not been used as a growth promoter since the early 1980s. Since VRE occur in sewage [16], it is likely that the bacteria can also be found in sewage sludge.

Antimicrobial resistance is a serious threat in human healthcare. It is thus important to maintain the low level of resistance among the Nordic food producing animals, which could spread the resistance genes to humans. Chicken consumption increases, and VRE do occur in low numbers in Swedish broiler chickens [19]. If sewage sludge is applied as a fertiliser to arable land, any VRE present could be spread in the environment, with a potential risk of entering food producing animals and thereby the human food chain. Moreover, resistance genes could be transferred to other bacteria in the environment. Novais et al., [20] and Iversen et al., [21] highlight the risk of spreading antibiotic resistant bacteria in the environment and of building up environmental reservoirs of antimicrobial resistance genes that might re-enter the ecosystem in human pathogenic bacteria.

The main aim of the present study was to investigate the occurrence of VRE in treated sewage sludge from a Swedish sewage treatment plant and to study their prevalence in the WWTP over time. A secondary aim was to compare VRE isolates from sewage sludge with isolates from humans and chickens.

## Methods

### Sampling in the wastewater treatment plant

Sewage sludge was sampled at the Kungsängen waste water treatment plant in Uppsala, Sweden, which serves 200 000 population equivalents. At the plant, sewage sludge is treated with mesophilic anaerobic digestion and dewatered by centrifugation. The site for sampling of dewatered sludge was at the sludge feeder after centrifugation. Samplings started 24 February 2003 and were repeated once a week for a total of 17 times during a period of more than four months, with the last sampling occasion on 30 June 2003. On every sampling occasion five (5) samples of approximately 200 g were taken during 1 hour with 12 minutes intervals from the sludge feeder (screw) after the centrifugation of the sewage sludge. On the first and fifth sampling occasions, only one sample was obtained due to technical problems at the plant. In total, 77 samples were collected and every sample was analysed separately. The treated and centrifuged sewage sludge had a dry matter content of 25–32% according to an electronic moisture analyser (Sartorius MA-30) at the WWTP. Samples were collected in clean disposable vessels and cultured within three hours at the National Veterinary Institute, Uppsala, Sweden.

### Bacteriological culture

Because of an expected low concentration of VRE in the sludge, samples were pre-enriched in enterococcosel broth (BBL, Baltimore, USA). Ten (10) g wet weight of sludge were mixed in a stomacher with 90 g of enterococcosel broth containing 8 mg/L vancomycin and 0.25 mg/L clindamycin. Clindamycin was added to improve the detection of VRE by reducing background flora. The concentration of clindamycin used (0.25 mg/L) is much lower than the MIC of enterococci and should not imply selection within the population of enterococci. After incubation at 37°C for 24 h, an aliquot of 0.1 mL from the pre-enrichment broth was plated on Slanetz-Bartley agar (Oxoid, UK) with 8 mg/L vancomycin (Sigma, Germany) and incubated at 37°C for 48 h.

In addition to the pre-enrichment procedure, direct culture was performed on the samples from sampling occasions 10–17. For this, aliquots of 0.1 mL were taken from the pre-enrichment broth before incubation (see above), plated on Slanetz-Bartley agar with 8 mg/L vancomycin and incubated at 37°C for 48 h. A total of 8 weeks of sampling (including 5 samples/week) gave 40 samples for direct plating.

### Confirmation and identification of VRE isolates

Colonies with morphology consistent with enterococci were sub-cultured on bile-esculin agar (Difco, Maryland, USA) and horse blood agar (LabM, Lancashire, UK) (37°C, 24 h). From each sample, 1–3 colonies with different morphology were chosen for further identification. Isolates with a positive reaction on bile-esculin agar and in PYR testing (pyrrolidonyl aminopeptidase) (Rosco, Denmark) were tested for antimicrobial susceptibility and identified to species level according to Devriese et al. [22] by use of the following biochemical tests: mannitol, sorbitol, arabinose, saccharose, ribose, raffinose and methyl-α-D-glucopyranoside.

Three isolates (SL26, SL63 and SL68), untypeable by biochemical tests, were 16S rRNA sequenced according to a procedure described by Johansson et al. [23].

In total, 84 suspected VRE strains were stored at -70°C for further analysis.

### Antimicrobial susceptibility testing

Antimicrobial susceptibility was tested by a microdilution method following the standards of the Clinical and Laboratory Standards Institute (CLSI) using VetMIC™ microdilution panels (National Veterinary Institute, Uppsala, Sweden). Antimicrobials and ranges tested are given in Additional file 1. In short, 4–5 colonies of a fresh overnight culture were diluted in 5 mL Muller-Hinton broth (Difco, Maryland, USA) and incubated at 37°C for 4 h. Ten microlitre of this suspension were diluted in 10 mL of Muller-Hinton broth and 50 μL of the diluted sample were inoculated into each well on a microtitre plate containing the dried antibacterial substances. The microtitre plate was then sealed with plastic tape and incubated at 37°C for 16–18 h. To check the purity, a droplet of the suspension was also streaked on blood agar, and incubated together with the microtitre plates. Analysis of the results was based on growth or no growth in the microtitre well. The MIC (Minimum Inhibitory Concentration) value was considered to be the lowest concentration of antibiotic that prevented bacterial growth.

PCR for detection of *vanA *and *vanB *genes was performed according to Dutka-Malen et al., [24]. *Enterococcus faecium *BM 4147 and *E. faecalis *V583 were used as positive control strains for *vanA *and *vanB *genes, respectively. Both strains were obtained from the Swedish Institute for Infectious Disease Control, Solna, Sweden.

### PhenePlate

The 84 VRE isolated from sewage sludge were subtyped by a rapid screening method for enterococci using PhP-FS microtitre plates (PhenePlate™, PhPlate Microplate Techniques AB, Stockholm, Sweden) [25]. The subtyping of enterococci on PhP-FS plates is based on biochemical tests with 24 different reagents, and was performed according to the manufacturer's instructions. In short, a few colonies were suspended in PhP-suspending media, inoculated onto the PhenePlates, and incubated at 37°C. The plates were read with an optical microplate reader connected to a computer after 16, 40 and 64 h. For the cluster analysis of the PhP-data, the unweighted-pair group method using average linkages (UPGMA) by the PhP-software (PhPlate Microplate Techniques AB) was used. Isolates with a similarity index greater than 0.975 were considered to be of the same PhP-subtype. The subtypes were named α, β, γ, δ and Φ (Additional file 1).

In addition, using the PhenePlate analysis, VRE isolates from sewage sludge were compared to other isolates. These were: three VRE isolated in 1998–1999 from sewage from the same WWTP as the sewage sludge [16]), three VRE isolated from chickens in 2005 (SVA, National Veterinary Institute, Uppsala, Sweden), two human isolates from SMI isolated 1997 and 2001 (Swedish Institute for Infectious Disease Control, Solna, Sweden), and five human VRE isolated in 2005 in Uppsala (UAS, The University Hospital, Uppsala, Sweden). The three isolates from chickens are of the same PhP and PFGE-type as the vast majority of VRE isolated from Swedish chicken within the framework of a monitoring program during years 2000 to 2005 [26]. The five human isolates from Uppsala were from one outbreak and considered identical when typed by PFGE [Torell E: The University Hospital, Uppsala, Sweden, 2006, personal communication].

### Pulsed Field Gel Electrophoresis (PFGE)

PFGE was performed according to a modified protocol based on Turabelidze et al. [27] to verify relationships of the PhP-subtypes. Two isolates from each PhP-subtype, most distant regarding time of isolation, were chosen for the PFGE. In addition, eight single subtype isolates from the pheneplate screening were analysed by PFGE.

Electrophoresis was performed using a CHEF DRII apparatus (BioRad Laboratories, Richmond Calif., USA). In the first block the initial switch time was 3.5 s, the final switch time was 25 s, and the run time was 20 h, and in the second block the initial switch time was 1 s and the final switch time 5 s for 16 h, at 6 V/cm. *Sma *I was the enzyme used for cleaving the DNA. Lambda Ladder PFG Marker (New England BioLabs Inc.) was used as a molecular size marker. Isolates were considered indistinguishable if the PFGE patterns were indistinguishable or differed by only one band.

## Results

### VRE isolates in sludge

**Figure 1:**
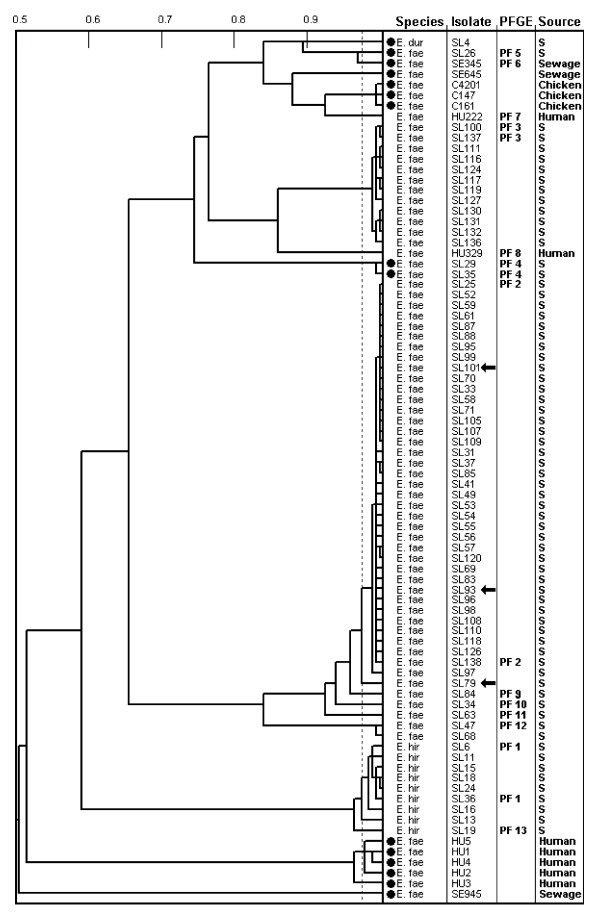
**Dendrogram showing UPGMA clustering of PhenePlate typing data for vancomycin resistant *E. faecium*, *E. hirae *and *E. durans *isolated from sludge (S)**. For comparison, vancomycin resistant *E. faecium *from sewage, chickens and humans are included. The horizontal axis of the dendrogram shows the similarities between isolates, and the dotted line indicates the identity level of 0.975. PFGE types are indicated for isolates tested by pulsed field gel electrophoresis (PFGE). Filled circles indicate isolates with vanA, the other isolates carried the vanB gene. Arrows indicate the isolates from direct culture.

After enrichment, VRE carrying the *vanA *or *vanB *genes were isolated from sludge samples on each of the 17 once-weekly sampling occasions. Sixty-one (61) of the 77 samples cultured (79%) tested positive for VRE (Table 1). In contrast, VRE were isolated from only three of 40 samples after direct plating. Subtyping with the PhenePlate (PhP) system identified 66 isolates (out of 84 analysed), each representing a unique PhP-subtype from a sample (Figure 1). All three isolates from direct culture belonged to one PhP-subtype (Figure 1).

**Table 1:** Species (*E. hirae, E. durans *and *E*. *faecium*) and PhenePlate subtypes of enterococci isolated by enrichment culture during the study period.

**Sampling week (date)**	*E. hirae*		*E. durans*	*E. faecium*				
	subtype α	single	single	subtype β	subtype γ	subtype δ	sutype Φ	single

I (24.2)*	**1**		**1**					
II (4.3)	**5**	**1 **(SL19)						
III (10.3)	**1**			**1**				**1 **(SL26)
IV (18.3)	**1**			**2**		**2 **(SL29, SL35)		**1 **(SL34)
V (24.3)*				**1**				
VI (31.3)				**1**				
VII (7.4)				**4**			**1**	
VIII (14.4)				**5**				
IX (22.4)				**1**				**1 **(SL63)
X (28.4)				**3**			**1**	
XI (5.5)				**4**				**1 **(SL84)
XII (12.5)				**5**	**1**			
XIII (19.5)				**5**	**1**			
XIV (26.5)				**2**	**3**			
XV (3.6)				**1**	**2**			
XVI (24.6)					**3**			
XVII (30.6)				**1**	**2**			

The figures represent the number of positive samples (out of 5 samples) on the actual date of sampling. The subtypes were differentiated using UPGMA clustering of PhP data. Identification of single subtype isolates in brackets.

*Only one sample obtained

*E. faecium *was the most prevalent species 85% (56/66) followed by *E. hirae *14% (9/66) and *E. durans *1.5% (1/66) (Table 1). Three isolates (SL26, SL63 and SL68) with uncertain species identity as judged from biochemical tests were identified as E. faecium by 16S rRNA gene sequencing. The sequence similarity of SL26 to *E. faecium*, strain DSM20477^T ^(GenBank accession number AJ276355) was 100% and the sequence identity to strain DSM20477^T ^was 99.7%. The sequence similarity of SL63 and SL68 to strain DSM20477^T ^was 100% and sequence identity to strain DSM20477^T ^was 99.9%. However, the three isolates may also represent *Enterococcus lactis *(GenBank accession number DQ255948), because the sequence similarities to this species is also high (99.8–99.9%).

PCR analysis revealed four isolates to harbour the *vanA *gene (one *E. durans *and three *E. faecium*), whereas the *vanB *gene was confirmed in the remaining isolates (Figure 1). In addition, *E. gallinarum *harbouring neither *vanA *nor *vanB *genes was isolated from one sample (MIC for vancomycin = 8). The three isolates from direct plating were all *E. faecium vanB*.

### PhenePlate analysis

The dendrogram in Figure 1 illustrates the results of PhenePlate analysis of 69 sewage sludge(SL) samples (including three from direct plating) and 13 other VRE isolates: human(HU), sewage(SE) and chickens(C). None of the 13 isolates of other origin belonged to the same PhP-subtype as the isolates from sludge samples (Figure 1).

The PhenePlate analysis showed that phenotypically identical strains were repeatedly isolated for up to 16 weeks. This observation was strengthened by indistinguishable PFGE patterns of isolates, most distant regarding time of isolation, within each PhP-subtype (Figure 1).

### Antibiograms

Antibiograms of isolates within a PhP-subtype were mostly similar and to some extent discriminatory between subtypes (Additional file 1). However, in *E. faecium *subtype β, five (5) isolates (SL33, SL37, SL69, SL70 and SL99) had divergent MIC values for gentamicin; MIC = 8 instead of MIC>512 as in the other 31 isolates. These five isolates were found in weeks IV to XII, i.e. over a nine-week period. In addition, one isolate (SL37) in the same subtype (*E. faecium *subtype β) had a MIC<16 to neomycin compared to the others, which had MIC values > 1024. One of the isolates from direct culture (SL93), which also belonged to the *E. faecium *subtype β, had a MIC value > 64 to tetracycline, whereas the other isolates had MIC values < 0.5–8.

## Discussion

VRE were isolated every week from sewage sludge during the four-month study. This indicates that VRE as well as enterococci survive mesophilic anaerobic digestion, as earlier shown by Sahlström et al 2004 [28]. Treatment with mesophilic digestion is a common way to treat sewage sludge in WWTPs in Sweden, which means that VRE may occur in sewage sludge in other parts of the country as well. The majority of the VRE isolated were *E. faecium *harbouring the *vanB *gene. This agrees with the fact that most VRE isolated from Swedish hospital patients are *E. faecium *carrying the *vanB *gene [17,29] but contrasts with the situation in other European countries, where *E. faecium vanA *is the most common cause of VRE infections [30].

**Figure 2:**
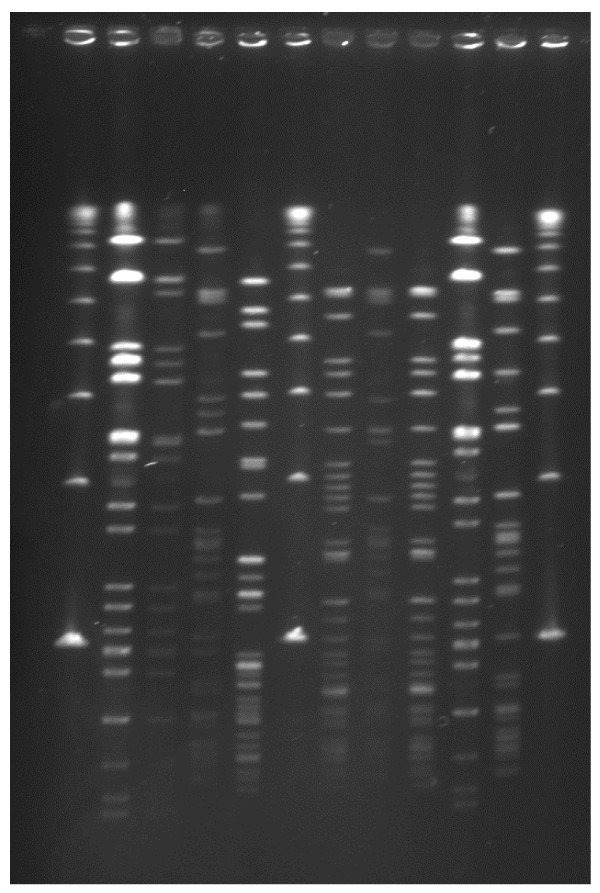
**PFGE patterns of sewage sludge samples (from left to right) λ, SL6, SL19, SL25, SL26, λ, SL29, SL34, SL35, SL36, SL47 and λ**. λ = Lambda Ladder PFG Marker, DNA size standard (New England BioLabs Inc.) Isolates SL6 and SL36 represent PFGE type 1 and isolates SL29 and SL35 represent PFGE type 4.

Isolation of VRE in Swedish healthcare is rare and as a consequence, inflow of VRE of human origin to WWTPs should be low [31]. The few human VRE strains analysed in this study differed regarding their PhenePlate profiles from the VRE isolates from sewage sludge. However, further comparisons of VRE from humans and sewage sludge would be interesting. The frequent isolation of VRE in this study could be due to the ability of enterococci (including VRE) to persist in the WWTP environment. PhenePlate analysis revealed that indistinguishable strains were repeatedly isolated from the sludge during a period of several weeks, a finding confirmed by PFGE analysis (figure 2). The longest interval between isolations was 15 weeks.

Bruinsma et al. [32] suggest a horizontal spread of the enterococcal *vanA *gene rather from pigs than from chickens to human strains. The source of human *E. faecium vanB *is not known, but could be another animal reservoir [33]. Our findings from sewage sludge, which can be assumed to reflect the human population, give no indication that human VRE in Sweden derive from animals, since we isolated mainly *E. faecium vanB*, whereas VRE in Swedish chickens are *E. faecium vanA *[26]. Moreover, VRE of the dominant PhP-subtype isolated from Swedish chickens were not found in sewage sludge (Figure 1), indicating that human colonisation with VRE from Swedish chickens is rare.

Our analysis illustrated a good correlation between the PhenePlate analysis (dendrogram) and the PFGE analysis (Figure 1). PhenePlate analysis has previously been found to be less discriminatory for *E. faecium *than for *E. faecalis *[34]. The *E. faecium *isolates, indistinguishable by UPGM clustering of PhenePlate-data (identity level = 0. 975), were likewise indistinguishable by PFGE, whereas less similar isolates (similarity level = 0.969) differed on PFGE (e.g. SE345 vs. SL26). Likewise, the antibiograms also discriminated PhP-subtypes from each other (Additional file 1).

The direct platings revealed three samples positive for *E. faecium *out of 40 samples cultured (7.5%). This is in contrast to the high isolation frequency after selective enrichment (79.2%), which appears to be the preferred procedure for isolation of VRE from sewage sludge. The lower isolation frequency after direct plating was probably due to the fact that VRE constitute a small proportion of enterococci in sewage sludge. In sewage from Spanish WWTPs, VRE were detected in low proportions, i.e. approximately 1:5000 (0.05%) of the total enterococci count, using pre-enrichment in enterococcosel broth [35]. In addition, Ferreira da Silva et al. [36] reported no VRE without pre-enrichment of sewage from a WWTP serving 100 000 inhabitants in Portugal. However in another Portuguese study, 3.1% VRE were isolated from a larger WWTP in the district capital Coimbra by direct plating, but the authors expected that findings would have been higher if an enrichment procedure had been used [37].

All of the vancomycin resistant *E. faecium *isolates carrying the *vanB *gene had high MICs to ampicillin (32->32 mg/L). This is in accordance with a described genetic link between ampicillin resistance and vancomycin resistance type *vanB2*, which is a subtype of *vanB *[38-40]. In contrast, the three *E. faecium vanA *isolates from sewage sludge had substantially lower MICs to ampicillin (0.5–1 mg/L).

In addition to *E. faecium*, other species of *Enterococci *isolated from sewage sludge were found to have high MICs to vancomycin. One isolate of *E. durans *from the first sampling of sludge had high level vancomycin resistance (MIC >128 μg/mL) and harboured the *vanA *gene. Torres et al. [41] were the first in the world to report *E. durans vanA *in sewage, but such strains have also been reported in sewage exposed to vancomycin waste [11]. Gambarotto et al. [42] isolated one *E. durans vanA *from pork meat in France, while Jenney et al. [43] reported isolation of *E. durans vanB *from a human patient. *Enterococcus gallinarum*, also isolated in our study, usually has an intrinsic low level resistance against vancomycin, mediated by *vanC *[10]. However, there are a few reports of *E. gallinarum *carrying *vanA *or *vanB *[44,45]. The isolation of *E. durans, E. gallinarum *and *E. hirae *in addition to *E. faecium *demonstrates that there is a broad spectrum of different species of enterococci carrying vancomycin resistance genes in sewage sludge. These species are not all considered to be especially pathogenic, but the main risk is in their possibility to transfer their resistance genes (except the intrinsic resistance in *E. gallinarum*) to other more pathogenic bacteria.

## Conclusion

In conclusion, sewage sludge contains vancomycin resistant enterococci, implying a potential risk of spreading VRE and resistance genes to the environment and possibly to the human and animal population. The frequent occurrence of VRE in mesophilically digested sewage sludge implies a need for more efficient hygienic treatment of sewage sludge, in order to avoid possible spread of antimicrobial resistance through use of sewage sludge on arable land. Usage of sewage sludge may contribute in spreading resistant bacteria by building up environmental reservoirs of antimicrobial resistance genes that enter the ecosystem, if there is not efficient hygienic treatment of sewage sludge before use [29,46].

## Competing interests

The authors declare that they have no competing interests.

## Authors' contributions

The study was designed by all authors. LS did the field work and the analysis was done by LS, VR and AAS. LS drafted the manuscript and all authors revised, read and approved the final manuscript.

## Supplementary Material

Additional file 1 **Minimum inhibitory concentration (MIC) for VRE per PhP subtype**. Only isolates after enrichment and only one isolate of each subtype per sample are shown. Click here for file
