# Supplementary material for: Vancomycin resistant enterococci (VRE) in Swedish sewage sludge
Source: Acta Vet Scand. 2009 May 29;51(1):24. doi: 10.1186/1751-0147-51-24 (PMC2693504; doi:10.1186/1751-0147-51-24)
Supplement: Additional file 1 — Minimum inhibitory concentration (MIC) for VRE per PhP subtype. Only isolates after enrichment and only one isolate of each subtype per sample are shown. [file 1751-0147-51-24-S1.doc]

Minimum inhibitory concentration (MIC) for VRE per PhP subtype. Only isolates after enrichment and only one isolate of each subtype per sample are shown.

| **Species** | **subtype** | **PCR** | **n** | **MICs**^*^ (mg/L)) | | | | | | | | | | | | |
| --- | --- | --- | --- | --- | --- | --- | --- | --- | --- | --- | --- | --- | --- | --- | --- | --- |
|  |  |  |  | **Am** | **Em** | **Vi** | **Tc** | **Gm** | **Nm** | **Sm** | **Cm** | **Van** | **Av** | **Na** | **Ba** | **Fl** |
|  | | |  | (0.25-32) | (0.5-64) | (0.5-64) | (0.5-64) | (0.5-512) | (2-1024) | (32-1024) | (2-16) | (1-128) | (0.5-64) | (0.12-16) | (1-64) | (2-128) |
| *E. hirae* | α | *vanB* | 8 | 2 | >64 | 2-4 | >64 | 8-16 | 8-32 | >1024 | 4-8 | 8-32 | 2-8 | 0.5-1 | <1-2 | >128 |
| *E. hirae* | single | *vanB* | 1 | 2 | >64 | 4 | >64 | 4 | 4 | >1024 | 8 | 8 | 2 | 2 | <1 | >128 |
| *E. durans* | single | *vanA* | 1 | 4 | >64 | 8 | >64 | 4 | 16 | >1024 | 16 | >128 | 4 | 0.5 | 16 | >128 |
| *E. faecium* | γ | *vanB* | 12 | >32 | >64 | 4-8 | <0.5-1 | 4-8 | >1024 | >1024 | 8-16 | 32->128 | 2-16 | 0.5-1 | 32-64 | >128 |
| *E. faecium* | β | *vanB* | 36 | 32->32 | >64 | 2-8 | <0.5-8 | 8**->512 | >1024*** | 1024->1024 | 4-16 | 64->128 | 1-8 | 0.5-1 | 16-32 | >128 |
| *E. faecium* | δ | *vanA* | 2 | 1 | >64 | 64 | >64 | 8 | >1024 | >1024 | 8 | >128 | 2-4 | 0.5 | 32 | >128 |
| *E. faecium* | ф | *vanB* | 2 | >32 | >64 | 2-4 | <0.5-1 | >512 | >1024 | >1024 | 8 | >128 | 2 | 0.5 | 32 | >128 |
| *E. faecium* | single | *vanA* | 1 | 0.5 | >64 | 64 | >64 | 8 | 32 | 1024 | 16 | >128 | 1 | 0.5 | 64 | >128 |
| *E. faecium* | single | *vanB* | 3**** | >32 | >64 | 4 | 0.5-2 | 8->512 | >1024 | >1024 | 8->16 | >128 | 1-2 | 0.5 | 16-32 | >128 |

* Am: ampicillin; Av: avilamycin; Ba: bacitracin; Cm: chloramphenicol; Em: erythromycin; Gm: gentamicin; Na: narasin; Nm: neomycin; Sm: streptomycin; Tc: tetracycline; Van: vancomycin; Vi: virginiamycin. Range of concentrations tested for each antimicrobial is given inside brackets.

** Four isolates had MIC=8, all the others in subtype β had MIC>512.

*** One isolate (37) had MIC=16, all the others in subtype β had MIC>1024.

**** Three samples had the same MIC values but different PhenePlate-subtypes (single).
